# Medical Care Use Among Patients with Monogenic Osteoporosis Due to Rare Variants in *LRP5*, *PLS3*, or *WNT1*

**DOI:** 10.1007/s00223-023-01101-3

**Published:** 2023-06-06

**Authors:** S. J. E. Verdonk, S. Storoni, L. Zhytnik, W. Zhong, G. Pals, B. J. van Royen, M. W. Elting, A. Maugeri, E. M. W. Eekhoff, D. Micha

**Affiliations:** 1 Department of Internal Medicine Section Endocrinology, Amsterdam UMC Location Vrije Universiteit Amsterdam, De Boelelaan 1117, Amsterdam, The Netherlands; 2 Rare Bone Disease Center Amsterdam, Amsterdam, The Netherlands; 3 Amsterdam Movement Sciences, Tissue Function and Regeneration, Amsterdam, The Netherlands; 4 Department of Human Genetics, Amsterdam UMC Location Vrije Universiteit Amsterdam, De Boelelaan 1117, Amsterdam, The Netherlands; 5 Department of Traumatology and Orthopedics, University of Tartu, Tartu, Estonia; 6 Department of Orthopedic Surgery and Sports Medicine, Amsterdam UMC Location University of Amsterdam and Location Vrije Universiteit Amsterdam, Meibergdreef 9, Amsterdam, The Netherlands; 7 Amsterdam Movement Sciences, Musculoskeletal Health, Amsterdam, The Netherlands

**Keywords:** Monogenic osteoporosis, Early-onset osteoporosis, LRP5, PLS3, WNT1, Osteogenesis imperfecta

## Abstract

Pathogenic variants in the *LRP5*, *PLS3*, or *WNT1* genes can significantly affect bone mineral density, causing monogenic osteoporosis. Much remains to be discovered about the phenotype and medical care needs of these patients. The purpose of this study was to examine the use of medical care among Dutch individuals identified between 2014 and 2021 with a pathogenic or suspicious rare variant in *LRP5*, *PLS3*, or *WNT1*. In addition, the aim was to compare their medical care utilization to both the overall Dutch population and the Dutch Osteogenesis Imperfecta (OI) population. The Amsterdam UMC Genome Database was used to match 92 patients with the Statistics Netherlands (CBS) cohort. Patients were categorized based on their harbored variants: *LRP5*, *PLS3*, or *WNT1*. Hospital admissions, outpatient visits, medication data, and diagnosis treatment combinations (DTCs) were compared between the variant groups and, when possible, to the total population and OI population. Compared to the total population, patients with an *LRP5*, *PLS3*, or *WNT1* variant had 1.63 times more hospital admissions, 2.0 times more opened DTCs, and a greater proportion using medication. Compared to OI patients, they had 0.62 times fewer admissions. Dutch patients with an *LRP5*, *PLS3*, or *WNT1* variant appear to require on average more medical care than the total population. As expected, they made higher use of care at the surgical and orthopedic departments. Additionally, they used more care at the audiological centers and the otorhinolaryngology (ENT) department, suggesting a higher risk of hearing-related problems.

## Introduction

Osteoporosis is a common multifactorial disorder that affects approximately 18.3% of the world’s population [1]. However, in rare cases, osteoporosis can be hereditary due to mutations in a single gene (monogenic osteoporosis). In contrast to osteoporosis found in postmenopausal women and elderly males, patients with monogenic osteoporosis present fractures associated with decreased bone mineral density (BMD) at a younger age [2]. Due to the wide implementation of next generation sequencing, several monogenic defects causing early-onset osteoporosis have been identified in recent years [3, 4]. This study focuses primarily on pathogenic or rare suspicious variants in low-density lipoprotein receptor-related protein 5* (LRP5)*, plastin 3 (*PLS3)*, or proto-oncogene Wnt-1 (*WNT1)* as monogenic causes of osteoporosis [5].

When addressing monogenic osteoporosis, osteogenesis imperfecta (OI) cannot be omitted. Compared to the widely investigated phenotype of OI patients, little is known about the phenotype of *LRP5*, *PLS3*, or *WNT1* individuals [6]. OI is a syndromic form of monogenic osteoporosis mainly caused by pathogenic variants in collagen type I encoding genes or in genes responsible for collagen type I posttranslational processing [7, 8]. Although bone fragility is the predominant clinical manifestation of OI, it is typically accompanied by extraskeletal manifestations such as blue sclerae, dentinogenesis imperfecta, impaired hearing, and increased joint laxity. It is unknown if and to what extent patients with *LRP5*, *PLS3*, or *WNT1* pathogenic variants exhibit comparable extraskeletal manifestations. The *LRP5*, *PLS3*, and *WNT1* genes are not directly engaged in collagen type I production but are involved in other bone formation-related pathways [9]. In particular, both *WNT1* and *LRP5* are involved in the Wnt signaling pathway. This pathway is activated by the interaction of WNT1 with the Frizzled receptor and an LRP5/6 co-receptor. This binding triggers an intracellular signaling cascade that stimulates preosteoblastic replication, induces osteoblastogenesis, and inhibits osteoblastic and osteocytic apoptosis [9]. The pathophysiology of *PLS3* is still unclear, but it probably influences bone homeostasis by regulating osteoclast activity [10].

The *LRP5*, *PLS3,* and *WNT1* genes all encode proteins in which monoallelic pathogenic variants can significantly affect the BMD. Biallelic pathogenic variants in *WNT1* are known to cause a severe form of OI (MIM#615,220), while heterozygous pathogenic variants cause juvenile osteoporosis (MIM#615,221) [11]. In *LRP5,* loss-of-function pathogenic variants as well as gain-of-function pathogenic variants have been described [12]. Biallelic loss-of-function pathogenic variants have been shown to cause osteoporosis pseudoglioma syndrome (MIM#259,770), a disorder characterized by early-onset osteoporosis and complications in eye development [13] or exudative vitreoretinopathy (MIM#601,813), a milder eye disorder. A heterozygous loss-of-function *LRP5* pathogenic variant can predispose to juvenile osteoporosis or exudative vitreoretinopathy [12, 14, 15]. Both disorders show variability in expression and incomplete penetrance and may represent different manifestations of the same disorder. The heterozygous gain-of-function *LRP5* pathogenic variant causes increased bone mineral density (MIM#607,634, MIM#144,750) [16]. The gene is in addition associated with polycystic liver disease 4 (MIM#617,875), lacking bone phenotype. A *PLS3* pathogenic variant causes X-linked osteoporosis [10]. Males with *PLS3* pathogenic variants experience severe osteoporosis and fracture at a young age (MIM#300,910), while in females with heterozygous pathogenic variants clinical presentation can vary in severity [17, 18]. It appears that people with *LRP5* and heterozygous *WNT1* pathogenic variants do not have extraskeletal characteristics similar to those found in patients with OI [6]. With the exception of reported blue or gray sclerae, individuals with *PLS3* pathogenic variants do not display noticeable extra-skeletal characteristics; nevertheless, this has been studied only sparingly [19, 20].

Given that the role of *LRP5*, *PLS3*, and *WNT1* in the development of osteoporosis was only discovered 10–15 years ago, and the rarity of these pathogenic variants, their associated morbidity and prognosis are still largely undetermined. It remains unclear which level of multidisciplinary care is needed for these patients [21]. The aim of this study was to describe the use of medical care by patients with an *LRP5*, *PLS3,* or *WNT1* pathogenic or suspicious rare variant in the Netherlands. Additionally, the goal was to compare the medical data of these patients with the medical data of the overall Dutch population and the Dutch OI population [22].

## Methods

### Study Participants

All patients diagnosed with either a pathogenic or a rare suspicious variant in *LRP5*, *PLS3*, or *WNT1* in the national reference center for the molecular diagnosis of OI in the Amsterdam UMC (Amsterdam UMC Genome Database) between 2014 and 2021 were eligible for inclusion in this study. We used the term “rare suspicious variants” to refer to genetic variations that, according to the ACMG classification, cannot be classified as likely pathogenic or pathogenic as yet due to their rarity and recent discovery [23, 24]. However, these variants of unknown significance (VUS) are highly suspicious of being pathogenic based on molecular data, population data, and patient and family data. To ensure that only patients with an osteoporotic phenotype were included, patients with *LRP5* gain-of-function pathogenic variants were excluded from the study cohort. As biallelic pathogenic variants in *WNT1* are known to cause a severe form of OI, individuals with these variants were excluded. The Dutch OI population, which has been previously described and also comes from the Amsterdam UMC genome database, was used to compare individuals with *LRP5*, *PLS3*, or *WNT1* mutations to OI patients [22, 25].

### Data Extraction

From the Amsterdam UMC Genome Database, a cohort of 92 individuals with a pathogenic or rare suspicious variant in *LRP5*, *PLS3*, or *WNT1* was extracted. Patients were matched to the cohort of Netherlands Statistics (CBS). The CBS has anonymized Dutch population health care information. Upon request, healthcare authorities and academic institutions can access CBS data for research purposes. Patients’ ages and, if applicable, their ages at death were retrieved from the CBS database (available between 1995 and 2019). Additionally, data regarding hospital admissions, diagnosis treatment combinations (DTCs), outpatient clinic consultations, and drug use was extracted. Information on admission duration, admission type, hospital type, admission date, and medical specialty was extracted from hospitalization data (available between 2013 and 2019). In the Dutch healthcare system, DTCs are used for billing purposes [26]. A DTC care product is the entirety of consultations, examinations, and treatments that a patient receives on average at a hospital for a particular diagnosis. Included in the extracted data were the number of opened DTCs (available between 2013 and 2017) and the registered medical specialty. Information regarding the date, type and number of outpatient clinic consultations was retrieved (available between 2013 and 2019). Moreover, data concerning medicine use (in 2017) was extracted. This included data on the main anatomical drug group to which the prescribed medication belonged according to the Anatomical Therapeutic Chemical (ATC) classification [27]. The extracted data included first-line medication, which could have been prescribed by a general practitioner or a specialist; data on the duration of the prescription was not available.

### Data Analysis

Data is presented for the *LRP5, PLS3, WNT1* cohort and, where group sizes allow, categorized by the affected gene. Patients were divided into four age groups: 0 to 24, 25 to 44, 45 to 64, and 65 years and older. Subgroup age distributions were presented as median with 10th and 90th percentiles. Data on admissions, DTCs, outpatient clinic visits, and medication prescriptions were analyzed descriptively and reported as absolute numbers and percentages, or as mean, median, and standard deviation.

Using incidence rate ratios (IRRs), the number of hospital admissions (including both day-case and inpatient admissions) and DTCs for patients with an *LRP5*, *PLS3*, or *WNT1* variant was compared to that of the whole population [28]. The CBS website provides free public access to information on the number of hospital admissions and DTCs in the Dutch population each year [29, 30]. In addition, the number of hospital admissions was compared to the number of admissions in the Dutch OI population [25]. The numerator of the IRRs was the mean number of admissions or DTCs per year in the *LRP5*, *PLS3*, and *WNT1* cohort, while the denominator was the mean found in the total population or the OI population. Since the age groups in this study were not the same as those in our previous study on how often OI patients used medical care [22], hospitalization rates for the OI population were calculated for the aforementioned age groups.

The percentage of patients who use medication was calculated for different age groups and compared to the percentage of people who use medication in the total population. Information on the percentage of the total Dutch population that uses medication is available to the general public [31]. The percentage of patients using medication was also investigated per anatomical main group. Medication usage for the following main groups was investigated and categorized per age group: ‘alimentary tract and metabolism’, ‘blood and blood forming organs’, ‘cardiovascular system’, ‘musculo-skeletal system’, ‘nervous system’, ‘respiratory system’ and ‘sensory organs’.

To ensure patient confidentiality, information regarding patient groups lower than 10 are generally not shown. Groups with fewer than 10 patients are only shown when they cannot be directly traced back to a person. Only descriptive statistical analyses were conducted. Since only the publicly available medical data on the total population could be used (which did not include the actual data of all individuals in the Netherlands), it was not possible to utilize inferential statistics to examine possible differences between patients with an *LRP5*, *PLS3*, or *WNT1* variant and the total population. Statistical analyses were performed by authors SJEV and SS using IBM SPSS Statistics for Windows version 25 (IBM corporation, Armonk, NY, USA).

### Ethical Consideration

The Medical Ethics Review Committee (MERC) of the Amsterdam UMC (Amsterdam, The Netherlands) waived the need for ethics approval and the need to acquire consent for the analysis and publishing of the retrospectively obtained and anonymized data for this non-intervention study (MERC study number 2021.0085).

## Results

**Fig. 1:**
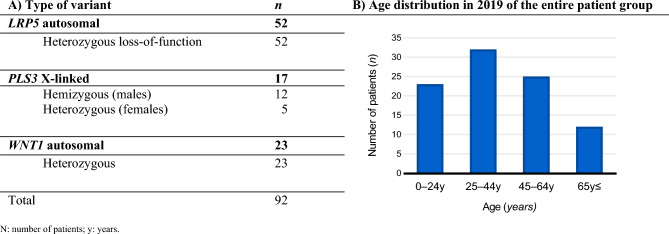
Characteristics of patients identified between 2014 and 2021 with *LRP5*, *PLS3* or *WNT1* variants in the Amsterdam UMC Genome Database

The Amsterdam UMC Genome Database comprised 92 individuals genetically identified with an *LRP5*, *PLS3*, or *WNT1* pathogenic or suspicious rare variant. Figure 1 shows the variant types and the age distribution as of January 2019. Of the 92 patients, 90 (97.8%) were matched with the CBS cohort. 52 patients had an *LRP5* variant, 15 had a *PLS3* variant, and 23 had a *WNT1* variant. In 2019, the median age and 10th and 90th percentiles were 40.2 years [13.0–68.4 years] for the entire group, 37.5 years [13.4–69.8 years] for patients with an *LRP5* variant, 47.0 years [10.7–63.6 years] for patients with a *PLS3* variant, and 45.5 years [20.2–75.07 years] for those with a *WNT1* variant. The median age at the time of genetic testing was 40.4 years [11.5–69.4 years].

Between 2013 and 2019, a total of 200 admissions took place, including both day-case and inpatient admissions. Patients with an *LRP5*, *PLS3*, or *WNT1* variant averaged 0.32 hospitalizations per person per year (median 0.14, SD ± 0.45). Table 1 presents the mean yearly admission rates for various age groups and the IRRs comparing patients with an *LRP5*, *PLS3*, or *WNT1* variant to the Dutch total population and the OI population. Patients with an *LRP5*, *PLS3*, or *WNT1* variant had, on average, 1.63 times more admissions than the total population and 0.62 times fewer admissions than the OI population. 40.0% (*n* = 36) of all patients had no admissions, 16.7% (*n* = 15) had one, 28.9% (*n* = 26) had 2 to 4, and 14.4% (*n* = 13) had five or more. The average length of hospital stay was 1.3 days (median 0.0; SD ± 3.1). 163 (81.5%) of the total 200 admissions were for non-acute care, while 37 (18.5%) were for acute care. There were 170 admissions to university medical centers or top clinical centers, compared to 30 admissions to general hospitals (respectively 85.0% and 15%). Of the 200 admissions, 80 concerned patients between 0 and 24 years old, 29 patients between 25 and 44 years old, 52 patients between 45 and 64 years old, and 39 patients 65 and older (respectively 40.0%, 14.5%, 26.0%, and 19.5%). Pediatrics accounted for 54 admissions (27%) while surgery and orthopedics accounted for 77 admissions (38.5%) combined.

**Table 1:** Admission incidence rate comparing the number of hospitalizations of Dutch patients with a pathogenic or a rare suspicious variant in *LRP5*, *PLS3* or *WNT1* to the total population and OI population between 2013 and 2019

**Mean yearly admission incidence rate on average per person**	**0–24 y**	**24–44 y**	**45–64 y**	** ≥ 65 y**	**Total**
*LRP5*, *PLS3*, or *WNT1* cohort	0.49	0.13	0.31	0.53	0.32
Total population [28, 29]	0.10	0.12	0.20	0.44	0.20
OI population [25]	0.81	0.25	0.33	NA*	0.51
**Incidence rate ratios (IRR)**	**0–24 y**	**24–44 y**	**45–64 y**	**≥ 65 y**	**Total**
*LRP5*, *PLS3*, or *WNT1* cohort compared to the total population	4.81	1.04	1.58	1.22	1.63
*LRP5*, *PLS3*, or *WNT1* cohort compared to OI population	0.60	0.52	0.94	NA*	0.62
**Mean yearly admission incidence and IRRs**	***LRP5***	***PLS3***	***WNT1***		
Mean yearly admission incidence rate	0.24	0.40	0.44		
IRR compared to the total Dutch population	1.24	2.05	2.23		
IRR compared to Dutch OI cohort	0.47	0.78	0.85		

Admissions include both day−case and inpatient admissions

*IRR* incidence rate ratio, *NA* not available, *y* years

* Since the Dutch OI population consisted of a relatively low number of patients 80 years and older, the ≥65 y age group could not be calculated accurately

The total number of DTCs opened for our patient cohort between 2013 and 2017 was 1002. 86.6% (*n* = 868) were opened in outpatient clinic settings, 7.0% (*n* = 70) during day-case admissions, and 5.6% (*n* = 56) during inpatient admissions. Table 2 presents the IRRs for the number of DTCs opened in patients with a pathogenic or a rare suspicious variant in *LRP5*, *PLS3*, or *WNT1* relative to the total Dutch population. The IRRs for the number of DTCs for OI patients relative to the total Dutch population are also provided in Table 2. Patients with an *LRP5*, *PLS3*, or *WNT1* variant had, on average, two times as many DTCs opened as the total Dutch population. Patients with an *LRP5*, *PLS3*, or *WNT1* variant had more DTCs opened for clinical genetics (IRR: 17.00), medical revalidation (IRR 7.68), pediatrics (IRR 4.78), orthopedics (IRR 4.37), rheumatology (IRR 3.86), the audiology center (IRR 3.58), internal medicine (IRR 2.63), and surgery (IRR 2.38) than the total Dutch population. Patients with an *LRP5*, *PLS3*, or *WNT1* variant had slightly more otorhinolaryngology DTCs opened than the entire Dutch population (IRR 1.85).

**Table 2:** Incidence ratio of the number of DTCs per person for patients with a pathogenic or a rare suspicious variant in *LRP5*, *PLS3* or *WNT1* compared to the total Dutch population during 2013–2017

Medical specialty for which a DTC was opened:	Total	*WNT1*	*LRP5*	*PLS3*	OI population [25]
Total	2.01	2.31	1.82	2.22	2.63
Pediatrics	4.78	2.62	5.03	7.19	9.90
Surgery	2.38	2.69	2.06	3.01	2.88
Plastic surgery	2.04	3.79	1.85	*NA*	1.72
Orthopedics	4.37	4.34	4.49	3.99	7.38
Internal medicine	2.63	2.86	2.60	2.37	1.74
Ophthalmology	1.02	1.28	0.67	1.85	1.07
Otorhinolaryngology	1.85	2.43	1.44	2.41	2.15
Urology	1.46	3.03	0.45	2.56	0.79
Obstetrics and gynecology	1.70	3.14	1.26	0.99	1.61
Gastroenterology	0.51	0.64	0.48	*NA*	0.71
Cardiology	0.57	1.27	0.42	*NA*	0.59
Pulmonology	0.63	1.66	0.36	*NA*	2.27
Neurology	0.99	0.89	0.96	1.26	1.19
Anesthesiology	2.75	3.95	3.02	*NA*	1.60
Dermatology	0.87	0.64	1.01	0.76	0.85
Rheumatology	3.86	4.07	2.91	6.79	0.80
Revalidation	7.68	6.13	7.16	11.85	20.90
Clinical genetics	17.00	12.17	16.35	26.67	25.82
Audiology center	3.58	6.07	2.05	5.04	7.54

Numbers present the incidence rate ratios calculated by dividing the mean number of DTCs per person in the LRP5, PLS3, and/or WNT1 group by the mean number of DTCs per person in the total Dutch population [28, 30] If the number of DTCs was based on less than three patients, the numbers are not shown (NA).

*DTC* diagnosis treatment combination, *NA* not avaiable

**Fig. 2:**
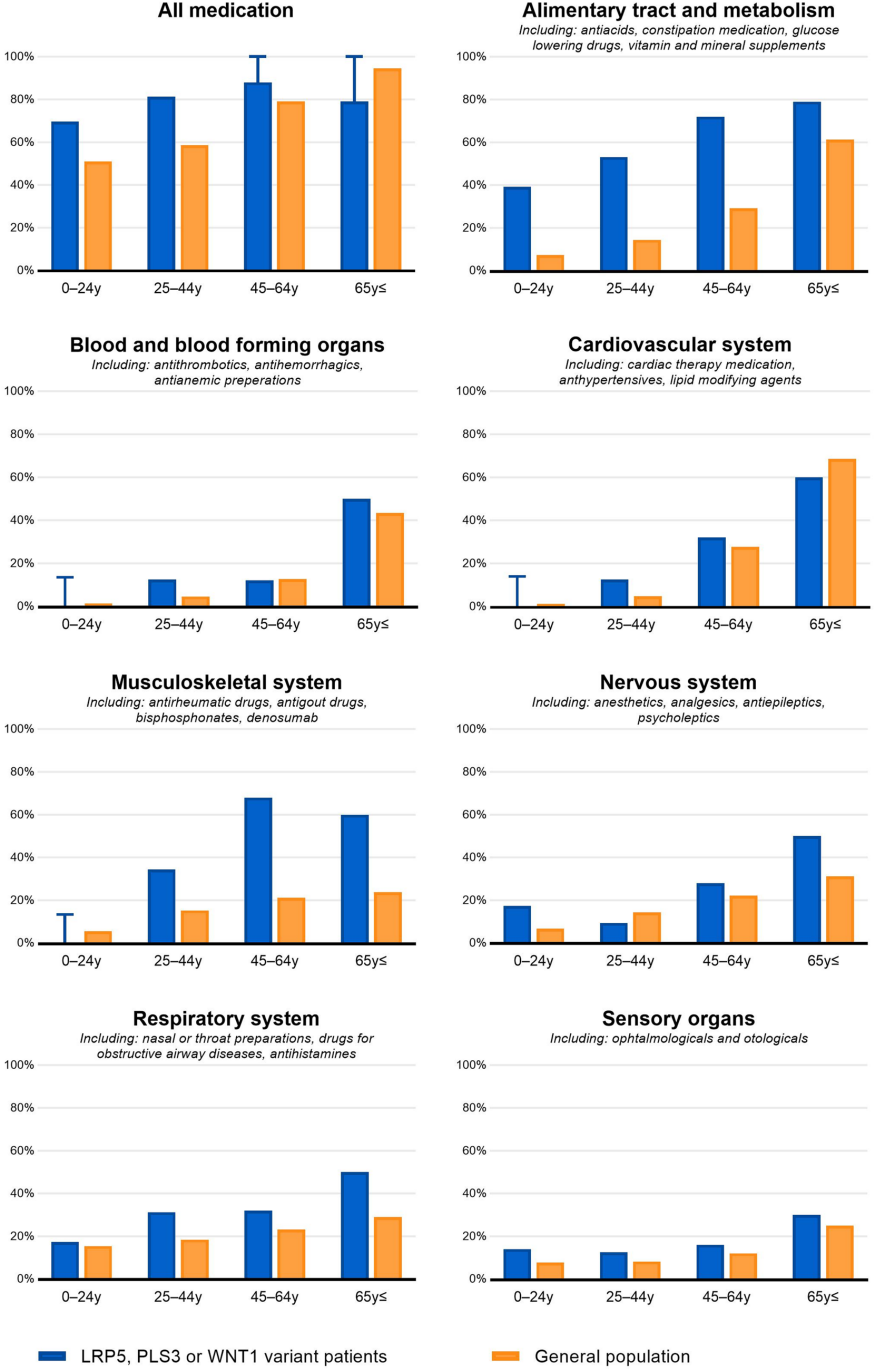
The proportion of people using medication according to ATC main groups; comparing patients with a pathogenic or a rare suspicious variant in *LRP5*, *PLS3*, or *WNT1* to the total Dutch population. Due to patient privacy, absolute numbers for certain categories cannot be shown; this is indicated by capped lines. For patients 0–24 years old, values ≤14% are not shown; for patients 45–64 years old, values ≥88% are not shown; for patients 65 years and older, values ≥79% are not shown. Y, years. For more information on ATC main groups the ATC/DDD index of the WHO Collaborating Centre for Drug Statistics Methodology can be consulted [27]. ATC, Anatomical Therapeutic Chemical; DDD, defined daily dose; WHO, World Health Organization.

In 2017, the average number of prescriptions per patient was 3.8 (median 3.0; SD ± 3.6). For patients with an *LRP5* variant, the mean number of drugs was 3.5 (median 2.5; SD ± 3.7); for patients with a *PLS3* variant, it was 4.1 (median 4.0; SD ± 3.6); and for patients with a *WNT1* variant, it was 4.1. (median 4.0; SD ± 3.4). Figure 2 displays the percentages of patients and the Dutch population as a whole who use medication, as well as for various ATC categories. For the age categories 0 to 24 years, 25 to 44 years, and 45 to 64 years, the proportion of patients with a variant in *LRP5*, *PLS3*, or *WNT1* using medication was higher compared to the total Dutch population (0–24 years: 69.6% vs. 51.0%; 25–44 years: 81.3% vs. 58.6%; 45–64 years: > 88% vs. 73.3%).

## Discussion

The aim of the current study was to investigate the use of medical care among 92 Dutch patients genetically diagnosed with a pathogenic or a rare suspicious variant in *LRP5*, *PLS3*, or *WNT1* between 2014 and 2021. Their use of medical care was compared to that of the entire population and of OI patients in the Netherlands.

In our cohort, patients with variants in *LRP5*, *PLS3,* or *WNT1* appeared to use more medical care compared to the total population (1.63 times more hospital admissions, 2.0 times more DTCs opened, and a larger proportion of people using medication, as shown in Fig. 2). Compared to the same age group in the total population, patients aged 0 to 24 experienced 4.81 times as many hospital admissions, and DTCs were opened 4.78 times more often for the pediatrics department. This is in agreement with the known onset of osteoporosis at childhood by *LRP5*, *PLS3*, and *WNT1* pathogenic variants [32]. Patients of all ages had more surgery and orthopedics DTCs than the total population (IRR: 2.38 and 4.37, respectively). Since *LRP5*, *PLS3*, and *WNT1* pathogenic variants cause osteoporosis, this was expected. This most likely also explains the relatively greater use of medication for ATC categories (‘alimentary tract and metabolism’ and ‘musculoskeletal system’) involving medications such as vitamin D, calcium, and bisphosphonates (Fig. 2).

When compared to the total population, patients with an *LRP5* variant appeared to have a similar or lower number of DTCs opened for the ophthalmology department (IRR 0.67). Since patients with loss-of-function *LRP5* variants may develop exudative vitreoretinopathy, a disease with a highly variability in phenotypic expression, we had anticipated that those with an *LRP5* variant would have more DTCs opened for ophthalmology [14]. The results indicate that specialists are not automatically triggered to refer these patients for an ophthalmologic screening. Interestingly, patients with an *LRP5*, *PLS3*, or *WNT1* variant had more audiological DTCs and slightly more otorhinolaryngology DTCs than the total population (IRR 3.58 and 1.85, respectively). This suggests that they may be at a higher risk of developing hearing issues. While it is possible that some of these DTCs could be attributed to patients being examined more often due to a suspicious diagnosis of OI, it is worth noting that routine screening for hearing issues in OI patients without hearing complaints is being proposed but is not yet commonplace as no guideline for hearing testing in OI currently exists [33]. Hearing loss, whether conductive, mixed, or sensorineural, is common in OI patients [34, 35]. Causes of hearing loss in patients with OI include footplate fixation, fractures of the ossicles, and otosclerosis. Patients with *LRP5*, *PLS3*, or *WNT1* variants may have similar issues. A disruption in the Wnt signaling pathway may also directly influence the development of the inner ear [36, 37]. As hearing loss can induce social isolation and is often detected late, in patients with *LRP5*, *PLS3*, or *WNT1* variants hearing screening should be considered [38]. In patients with variants in *LRP5*, *PLS3*, or *WNT1*, there was a slightly higher incidence of patients using respiratory system drugs (Fig. 2). Although the exact pathophysiology is not clear, patients with OI may have respiratory problems [39]. Due to skeletal thoracic problems, patients with *LRP5*, *PLS3*, or *WNT1* variants may have a greater risk of respiratory complications [40]; however, drawing this conclusion solely based on medication prescription data is premature as it needs more investigation.

Despite the small sample size, patients with a variant in *PLS3* or *WNT1* appeared to use more medical care than patients with a variant in *LRP5* (yearly admission rates: 0.40, 0.44, and 0.24, respectively; IRR for DTCs 2.22, 2.31, and 1.82, respectively). To our knowledge, no study exists comparing patients with *LRP5*, *PLS3*, or *WNT1* variants, and only limited research on the characteristics of these patients is available [11–13, 18, 32, 41]. Despite the inclusion of heterozygote *PLS3* females, the apparently increased need for hospital care reported in *PLS3* patients appears to indicate that in this cohort, males with a *PLS3* variant have a more severe phenotype than patients with an *LRP5* variant. Additional information on the comorbidities of individuals with *LRP5*, *PLS3*, or *WNT1* pathogenic variants may aid in providing more information on the prognosis and, as a result, clinical management advice.

Patients with an *LRP5*, *PLS3*, or *WNT1* variant used medical care less frequently than OI patients (0.61 times fewer admissions). Relative to those with genetic variants that cause mild forms of OI, which our group has previously reported on, the admission rates appear similar [25]. Because the *LRP5*, *PLS3,* or *WNT1* patients in this study included heterozygous *PLS3* females, it is possible that the amount of hospital care used by hemizygous *PLS3* males is greater than what is reported. Experts are divided on whether osteoporotic pathogenic variants in the *LRP5*, *PLS3,* or *WNT1* genes should be classified as OI [2, 12]. Patients with OI present syndromic symptoms in addition to brittle bones, which are not present in osteoporotic patients. Nevertheless, apart from a potentially greater frequency of hearing-related disorders, the hospital data evaluated in our analyses revealed no other relevant syndromic traits.

This study cannot avoid a few limitations. Firstly, due to low patient numbers, all pathogenic and rare suspicious variants in *LRP5*, *PLS3*, or *WNT1* have been grouped. Subdividing into smaller groups was not possible as it could not ensure patient confidentiality. Although the medical care data used is quite elaborate, we did not possess any additional deep phenotyping data that could be linked to the medical care data. As a result, this limited our investigation to the presence (or absence) of extraskeletal characteristics. Due to the fact that pathogenic variants of *LRP5*, *PLS3*, or *WNT1* were not included in the routine genetic testing of our center until 2014, shortly after the discovery of these genes, it was not possible to estimate the prevalence of these variants in the Netherlands.

In conclusion, it appears that patients with a pathogenic or rare suspicious mutation in *LRP5*, *PLS3*, or *WNT1* use more medical care than the general population. This is the first study to present medical care data for a large cohort of individuals with an *LRP5*, *PLS3*, or *WNT1* variant. As anticipated, patients with an *LRP5*, *PLS3*, or *WNT1* variant were more likely to require surgical and orthopedic hospital treatment. Additionally, treatment by audiological centers and otorhinolaryngology departments was increased. This may be indicative of a higher prevalence of hearing-related issues; hearing tests should thus be considered as part of the clinical management. Further research is needed to determine whether people with *LRP5*, *PLS3*, or *WNT1* variants have similar medical care requirements as those with OI.

## Data Availability

The original contributions presented in the study are included in the article. Further inquiries can be directed to the corresponding author.

## Abbreviations

ATC: Anatomical Therapeutic Chemical
BMD: Bone mineral density
CBS: Statistics Netherlands
DTC: Diagnosis treatment combination
ENT: Ear, nose, throat
IRR: Incidence rate ratio
LRP5: Low-density lipoprotein receptor-related protein 5
MERC: Medical Ethics Review Committee
MIM: Mendelian Inheritance in Man
OI: Osteogenesis imperfect
PLS3: Plastin 3
VUS: Variant of uncertain significance
WNT1: Proto-oncogene Wnt-1
